# Complete Genome Sequence of a Novel Human WU Polyomavirus Isolate Associated with Acute Respiratory Infection

**DOI:** 10.1128/genomeA.00177-16

**Published:** 2016-05-05

**Authors:** Darrell L. Dinwiddie, Walter N. Dehority, Kurt C. Schwalm, Jesse M. Young, Stephen M. Gross, Gary P. Schroth, Stephen A. Young

**Affiliations:** a Department of Pediatrics, University of New Mexico Health Sciences Center, Albuquerque, New Mexico, USA; b Clinical Translational Science Center, University of New Mexico Health Sciences Center, Albuquerque, New Mexico, USA; c University of New Mexico Biomedical Sciences Graduate Program, Albuquerque, New Mexico, USA; d Illumina, San Diego, California, USA; e TriCore Reference Laboratories, Albuquerque, New Mexico, USA

## Abstract

We report here the complete genome sequence of a WU polyomavirus (WUPyV) isolate, NM040708, collected from a patient with an acute respiratory infection in New Mexico. The double-stranded DNA (dsDNA) genome of NM040708 is 5,229 bp in length and differs from the WUPyV reference with accession no. NC_009539 by 6 nucleotides and 2 amino acids.

## GENOME ANNOUNCEMENT

WU polyomavirus (WUPyV) was first described in 2007 when it was cloned from the respiratory tract samples from children suffering from acute respiratory infection (1). The WUPyV genome is a highly conserved double-stranded circular genome of approximately 5,530 bp (2, 3). The rate of seropositivity to WUpvV in the adult population is near 100% (4, 5), and approximately 50% of children are infected with WUPyV within the first 18 months of life (6), indicating the wide distribution of the virus. Despite the ubiquitous nature of WUPyV, the pathogenicity of the virus remains unclear. Some studies have reported clinical symptoms in only a subset of patients in whom an active infection is detected, even if it is their first infection with the virus (6). In other studies, virus detection and clinical symptoms are reported only in immunosuppressed patients (2, 7) or with coinfections (2, 6, 8). Here, we report the complete genome sequence of WUPyV isolate NM040708 collected from a patient with an acute respiratory infection in New Mexico.

Total RNA was isolated using the Zymo Direct-zol kit (Zymo, USA) from a nasopharyngeal swab collected from a patient with acute respiratory infection and placed in viral transport medium. A stranded RNA Illumina sequencing library was produced using the RNA Access protocol (Illumina, USA), according to the manufacturer’s recommendations. Viral RNA sequences were captured using the University of New Mexico (UNM) ResVir panel containing 5,683 hybridization probes of 80 nucleotides in length designed to be complementary to coding sequence (CDS) regions of 31 human respiratory viruses, including WUPyV. Sequencing was conducted in a paired manner using V3 sequencing chemistry and 2 × 75-bp read lengths on an Illumina MiSeq. The sequencing reads were aligned to reference sequences of the targeted respiratory viruses using CLC Genomics Workbench (Qiagen, USA). A total of 900,802 sequencing reads aligned to the 5,229-bp WUPyV reference sequence (accession no. NC_009539), resulting in a mean depth of 12,916× (minimum, 21×; maximum, 61,966×) and 100% coverage of the genome.

To obtain a complete genome sequence, we conducted both alignment-guided consensus sequence generation and *de novo* assembly. Both methods produced a genome of 5,229 bp, with 100% agreement. Previous complete genome sequencing of WUPyV has revealed a highly conserved genome with minimal genomic variation (2). In our sample, alignment to NC_009539 revealed a total of 6 variants, of which 2 were nonsynonymous. We detected a single-amino-acid-changing variant in the large T antigen, Ile594Leu (accession no. YP_001285488.1), and one in the VP2 protein, Glu250Gln (accession no. YP_001285485.1). Phylogenetic analysis was conducted using neighbor joining with 1,000 bootstraps to 64 compete WUPyV genomes using CLC Genomics Workbench. Isolate NM040708 grouped most closely with genotype 1A isolates B3571, B3655, and B4932 (NCBI nucleotide accession numbers GU296372, GU296373, and GU296376, respectively), which were all collected from Australia in 2003, and strain T38 (NCBI accession no. GU296399), which was collected in 2003 from Canada (2). At the nucleotide level, NM040708 and B3571 differed by a single base pair at genomic position 2146 (ADD50976.1: G477C). The substitution of a cytosine for a guanine at position 477 was not predicted to cause an amino acid change in the VP1 protein, suggesting that the two isolates encode identical proteins.

### Nucleotide sequence accession number.

The whole-genome sequence of isolate NM040708 has been deposited in GenBank under the accession no. KU672381.
